# Pulmonary intravascular blood volume changes through the cardiac cycle in healthy volunteers studied by cardiovascular magnetic resonance measurements of arterial and venous flow

**DOI:** 10.1186/1532-429X-11-42

**Published:** 2009-10-30

**Authors:** Martin Ugander, Erik Jense, Hakan Arheden

**Affiliations:** 1 Cardiac MR group, Department of Clinical Physiology, Lund University Hospital, 22185 Lund, Sweden

## Abstract

**Background:**

This study aims to present a novel method for using cardiovascular magnetic resonance (CMR) to non-invasively quantify the variation in pulmonary blood volume throughout the cardiac cycle in humans.

**Methods:**

10 healthy volunteers (7 males, 3 female, age range 21-32 years) were studied. The blood flow in the pulmonary artery and all pulmonary veins was quantified during free breathing using phase contrast velocity encoded CMR. The difference in flow between the pulmonary artery and the pulmonary veins was integrated to calculate the change in pulmonary blood volume throughout the cardiac cycle.

**Results:**

The stroke volumes in the pulmonary artery and the sum of the pulmonary veins were (mean ± SEM) 103 ± 6 ml and 95 ± 6 ml, respectively. The pulmonary blood volume variation (PBVV) was 48 ± 5 ml, and the PBVV expressed as percent of the pulmonary artery stroke volume was 46 ± 3%. The maximum increase in pulmonary blood volume occurred 310 ± 12 ms after the R-wave from the ECG (32 ± 2% of the cardiac cycle). PBVV did not correlate to change in cross-sectional area in the pulmonary artery (R2 = 0.03, p = 0.66).

**Conclusion:**

It is feasible to non-invasively quantify the change in pulmonary blood volume during the cardiac cycle in humans using CMR. The average pulmonary blood volume variation in healthy volunteers was approximately 50 ml and this was approximately 50% of the stroke volume. Further studies are needed to assess the utility of the pulmonary blood volume variation as a measure for identifying cardiac and pulmonary vascular disease.

## Background

The blood volume in the pulmonary vasculature has been shown to vary throughout the cardiac cycle. This ability of the pulmonary vessels to expand and harbor a greater blood volume during systole is dependent on the stiffness of the vessels. If the vessels are rigid and non-expansive, the blood volume increase will not be as great as in healthy vessels. It has been proposed that the blood volume increase in the pulmonary vasculature represents the difference between the flow in the pulmonary artery and the pulmonary veins [1], however this has not been demonstrated. As the right ventricle (RV) ejects blood in to the pulmonary circulation, blood returns to the heart via the pulmonary veins. Since the ejection of blood from the RV is more pulsatile than the return of blood to the heart [2], there will be a transitory increase in the pulmonary blood volume during systole [3,4]. How great this transitory blood volume increase will be is partly determined by the properties of the pulmonary vasculature. Changes in properties such as vascular resistance and compliance are established measures of vascular disease [4-6].

The ability of the blood vessels to expand decreases with age and the progression of vascular disease or hypertension. The pulmonary blood volume variation has been shown to decrease with increasing age [3]. This may be the result of the increased stiffness of the pulmonary vessels. Furthermore, patients with thalassemia have been shown to have a lower pulmonary blood volume variation than healthy subjects [1]. This relationship between the arterial compliance and pulmonary blood volume variation makes it desirable to develop an accurate method for quantifying the blood volume variation. Several different techniques have been reported. Methods used in prior studies include Nitrous oxide-body plethysmography [6], cardiac catheterization [4] and ECG-gated radionuclide blood pool scintigraphy [7].

Cardiovascular Magnetic Resonance (CMR) is a safe and non-invasive technique. It has been proven to have excellent accuracy and repeatability when measuring blood flow in large vessels [8,9]. With CMR, the blood flow in the pulmonary artery and the pulmonary veins can be measured separately. We therefore hypothesized that the difference in flow entering and exiting the pulmonary vasculature measured by CMR could be used to quantify the variation in pulmonary blood volume throughout the cardiac cycle.

## Methods

### Study population

The local research ethics committee approved the study and all subjects provided written informed consent. 10 healthy volunteers were included, 7 males and 3 females, age range 21-32 years, BMI range 19.7-29.1 kg/m^2^.

### MR Imaging

**Figure 1:**
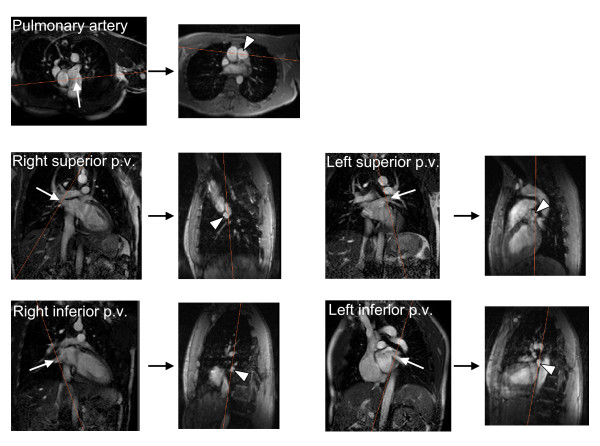
**Illustration of the imaging planes used to measure blood flow by through-plane velocity-encoded phase-contrast CMR in the pulmonary artery and each of the four pulmonary veins (p. v.) in a representative subject**. Five pairs of images connected by a black arrow are shown, one pair for each vessel. The left image in each pair is an image prescribed along the long-axis of the vessel and the white arrow indicates the direction of blood flow through the vessel. The right image is the modulus image for the velocity-encoded image prescribed perpendicular to the vessel, and the cross section of the vessel of interest is indicated by the arrow head. The orange lines in each image illustrate the intersection of the paired imaging planes, respectively.

All imaging was performed in the supine position using a 1.5T system (Intera, Philips, Best, the Netherlands) and a five element cardiac coil. Flow in the pulmonary artery and in the pulmonary veins was measured during free breathing using a fast field echo through-plane gradient echo phase contrast velocity encoded sequence in a plane positioned perpendicular to the respective vessels. The imaging planes for the respective vessels are illustrated in Figure 1. Typical imaging parameters for phase contrast imaging were: field of view 300 × 225 mm, matrix 128 × 96, acquired spatial resolution 2.3 × 2.3 × 6.0 mm, reconstructed spatial resolution 1.2 × 1.2 × 6.0 mm, flip angle 15 degrees, cardiac phases 35, temporal resolution 25 ms, velocity encoding gradient for arteries: 200 cm/s, veins: 80 cm/s, and retrospective gating, no breath holding, no parallel imaging, duration of acquisition 1 minute 37 seconds, number of signal averages 1, linear phase correction (LPC) filter on, Maxwell correction filter on, scanner software version number R11.1.2.

### Image Analysis

The blood flow entering and leaving the pulmonary vasculature was quantified. All MR image analysis was performed using commercially available software (Viewforum, Philips Medical Systems, Best, the Netherlands). Measurements were made throughout the cardiac cycle with the R-wave from the ECG as the starting point.

**Figure 2:**
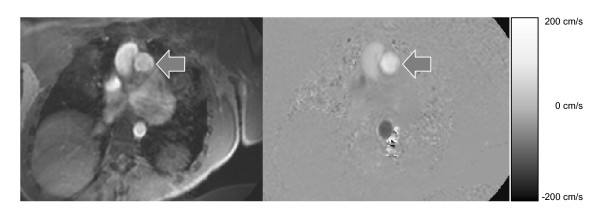
**The method for measuring flow in a vessel using cardiovascular magnetic resonance imaging**. The left panel shows the modulus reconstructed phase contrast image in a cross section of the pulmonary artery (arrow). The right panel shows the corresponding phase reconstructed image. The scale to the right shows the velocities in the through-plane direction. Figure 3A shows the corresponding flow in the pulmonary artery.

Flow in a vessel was determined by manually defining a region of interest (ROI) in the lumen of the vessel of interest using modulus reconstructed PC images (Figure 2). The average signal intensity in the same ROI in the phase reconstructed PC images was multiplied by the velocity encoding gradient and the area of the region of interest in order to determine instantaneous flow in ml/s at each time point. The result is a curve that displays the flow in a given vessel throughout the cardiac cycle (Figure 2).

### Calculating the cumulative change in blood volume in the pulmonary vasculature during the cardiac cycle

**Figure 3:**
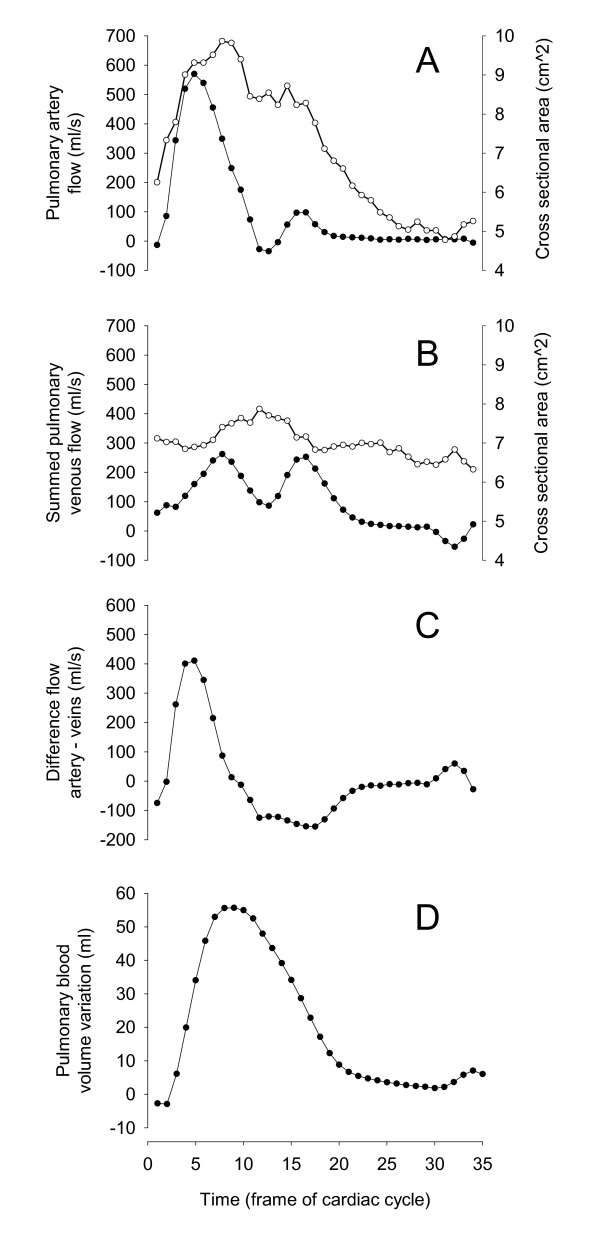
**The method to calculate the pulmonary blood volume variation**. Panels A and B show the instantaneous blood flow (filled circles) and cross sectional area (open circles) throughout the cardiac cycle in the pulmonary artery and in the sum of the pulmonary veins, respectively. Panel C shows the flow difference between the pulmonary artery and the sum of the pulmonary veins. Panel D shows the integral of the graph in Panel C, thus showing the cumulative pulmonary blood volume variation during the cardiac cycle.

Flow was measured both in the pulmonary artery (Figure 3A) and in all the pulmonary veins. The sum of the flow in all pulmonary veins represents the total outflow of blood from the pulmonary vasculature (Figure 3B). The sum of flow in the pulmonary veins was subtracted from the flow in the pulmonary artery (Figure 3C). The result is a graph showing the net flow of blood entering and leaving the pulmonary vasculature at any given time point during the cardiac cycle. The integral of this curve is the cumulative change in blood volume in the pulmonary vasculature during the cardiac cycle (Figure 3D). The difference between the maximum and minimum on this curve represents the maximum pulmonary blood volume variation (PBVV). The relative pulmonary blood volume increase was calculated by dividing the PBVV by the stroke volume in the pulmonary artery. To calculate the stroke volume in any given vessel the instantaneous flow throughout the cardiac cycle was integrated from the flow measurements. The R-R interval was recorded during the measurements and was used to calculate the heart rate and the time between the R-wave and the peak volume change called time of peak volume. The cross-sectional area (cm^2^) of the pulmonary artery and pulmonary veins was measured over the cardiac cycle.

### Statistics

Data are presented as mean ± standard error of the mean (SEM). Statistical comparison was performed with Wilcoxon's test. Linear regression analysis was performed with Pearson's linear correlation. P < 0.05 was considered statistically significant.

## Results

**Figure 4:**
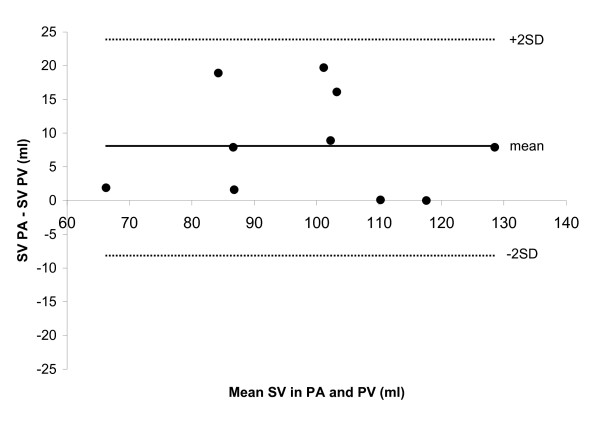
**Bland-Altman diagram illustrating the difference between the stroke volume (SV) in the pulmonary artery (PA) and the sum of the pulmonary veins (PV)**. Mean difference ± SD was 8 ± 8 ml (9 ± 8%).

Table 1 shows the subject characteristics and results obtained for the pulmonary blood volume variation. The stroke volume in the pulmonary artery and the sum of pulmonary veins was 103 ± 6 ml and 95 ± 6 ml, respectively (p = 0.008), and differed by 8 ± 8 ml (9 ± 8%) (Figure 4). The pulmonary blood volume variation was 48 ± 5 ml. The pulmonary blood volume variation expressed as percentage of SV in the pulmonary artery was 46 ± 3%. This occurred 310 ± 12 ms after the R-wave of the ECG (32 ± 2% of the cardiac cycle). The heart rate for acquisition of flow in the pulmonary artery and average heart rate for the acquisitions of flow in the pulmonary veins did not differ (63.1 ± 12.9 beats per minute vs. 62.7 ± 12.8 beats per minute, p = 0.38). There was no correlation between the change in cross-sectional area of the pulmonary artery and the pulmonary blood volume variation (R^2 ^= 0.03, p = 0.66).

**Table 1:** The Pulmonary blood volume variation in all subjects.

**Subject**	**Gender**	**Age (y)**	**PBVV (ml)**	**SV (ml)**	**PBVV (%SV)**
1	F	24	36	91	40

2	M	23	56	94	60

3	F	24	29	88	34

4	M	22	46	118	39

5	M	22	57	111	51

6	F	23	22	67	32

7	M	30	53	107	49

8	M	32	62	111	56

9	M	24	59	133	44

10	M	21	62	110	56

**Means ± SEM**		25 ± 1	48 ± 5	103 ± 6	46 ± 3

y = years, PBVV = pulmonary blood volume variation, ml = millilitres, SV = stroke volume in the pulmonary trunk, SEM = standard error of the mean

## Discussion

This study implemented a novel method of using velocity encoded CMR to quantify the change in pulmonary blood volume throughout the cardiac cycle. We found that the pulmonary blood volume increases on average just less than 50 ml during systole, which was just under 50% of the SV in the pulmonary artery.

The results found in this study differ from the results obtained in prior studies using other methods. A study using nitrous oxide-body plethysmography reported the relative blood volume increase to be 67% [6]. Values of 62% and 63% were obtained using cardiac catheterization and ECG gated radionuclide blood pool scintigraphy, respectively [5,7]. Both nitrous oxide-body plethysmography and catheterization methods measured only the pulmonary *arterial *blood volume variation [5,6]. These methods produce a larger blood volume variation due to the fact that there is a constant venous flow during the entire cardiac cycle which was not considered. Furthermore, measurements performed using cardiac catheterization showed a high variability [5], and nitrous oxide-body plethysmography method requires the use of a hyperbaric chamber, which does not have widespread availability. Moreover, the plethysmographic technique requires considerable subject cooperation which may affect the accuracy of the results [6]. ECG-gated radionuclide blood pool scintigraphy does consider the venous flow but is limited because it is a planar technique which has limited spatial resolution. Notably, neither the current or previous studies has studied the pulmonary blood volume variation in relation to the absolute pulmonary blood volume. Also, using the CMR method, the volume changes of the structurally and functionally distinct arterial, capillary and venous compartments remain unknown.

We found that the change in cross-sectional area of the pulmonary artery did not correlate with the pulmonary blood volume variation. Previous studies have used the change in cross sectional area of the pulmonary artery to assess the pulmonary vasculature in congenital heart disease [10]. Our findings indicate that the pulmonary blood volume variation is independent of this measure, and should thus not be seen as equivalent.

The results obtained in this study are based on determining stroke volume in different large vessels using velocity encoded CMR. This method is established as accurate and highly reproducible [2,8]. Furthermore, blood flow has previously been measured in all the vessels leading to and from the heart or pulmonary circulation [2,9,11]. The 8 ml (9%) lesser stroke volume in the sum of the pulmonary veins compared to the pulmonary trunk may be attributable to a small shunting of blood to the bronchial venous circulation and/or small measurement error when measuring the stroke volume as the sum of flow in four different vessels. A previous study showed a slightly greater stroke volume in the sum of the pulmonary veins compared to the pulmonary artery [9]. The source of the differences between that study and the current study are not known. One can only speculate that it might be related to differences in the post processing analysis by different observers. The cross-sectional area of the individual pulmonary veins in the subjects in the current study was on the order of 1.5 cm^2^. This would imply that approximately 5 × 5 pixels of acquired data and 10 × 10 pixels of interpolated data were included in the analysis. Notably, we have previously shown excellent interobserver variability for measurement of flow in such pulmonary veins [2].

The flow in the different vessels was measured serially and not simultaneously. However, heart rate did not change between acquisitions in the different vessels. Importantly, CMR has excellent agreement between measurements of change in volume derived from flow when compared to volumetric planimetry in the heart [2,11]. The accuracy of flow measurements by CMR is of paramount importance in a study such as this one. Studies have shown that newer CMR methods for measuring flow during a single breath hold may introduce considerable errors [12]. Thus, this study used non-breath hold techniques which are less susceptible to these errors. Motion blurring artefacts due to breathing were present, but not to an extent that imaging had to be repeated due to unacceptable image quality. Also, it has been shown that there is an approximately 20% variation in the stroke volume during free breathing [13], however, these differences would average out during the one and a half minute long acquisition in the current study.

The ability of the pulmonary vasculature to store blood during the pulsatile inflow of blood from the pulmonary artery is partly dependent on how much the vessels in the lung can dilate. It is also affected by the afterload which must be overcome when blood exits the pulmonary vasculature to the left atrium, e.g. the left atrial pressure. Hence, reduced compliance will result in reduced relative blood volume increase in the pulmonary vasculature. Reduced compliance is associated not only with increasing age but also with vascular disease and persistent hypertension [14,15]. It would therefore be of interest to determine the arterial compliance in the effort to identify vascular disease. It would also be of interest to study populations with vascular disease and pulmonary hypertension in comparison to matched healthy controls.

Furthermore, the values presented in the current study are limited to the age group studied and the supine body position, and it might be of value to study the pulmonary blood volume variation in a population with a larger age distribution, and explore the effect of different body positions on the pulmonary vasculature.

## Conclusion

It is feasible to non-invasively quantify the change in pulmonary blood volume during the cardiac cycle in humans using CMR. The average pulmonary blood volume variation in healthy volunteers was approximately 50 ml and this was approximately 50% of the stroke volume. Further studies are needed to assess the utility of the pulmonary blood volume variation as a measure for identifying cardiac and pulmonary vascular disease.

## Abbreviations

(PBVV): Pulmonary blood volume variation; (CMR): cardiovascular magnetic resonance; (RV): right ventricle; (SV): stroke volume; (T): Tesla.

## Competing interests

The authors declare that they have no competing interests.

## Authors' contributions

MU conceived of and designed the study, performed data acquisition, data analysis, statistical analysis and drafted the manuscript. EJ performed data analysis and drafted the manuscript. HA designed the study and helped to draft the manuscript. All authors read and approved the final manuscript.
